# Share – Workpackage 4: addressing the need for health care for paediatric rheumatic diseases throughout europe

**DOI:** 10.1186/1546-0096-12-S1-P136

**Published:** 2014-09-17

**Authors:** Pavla Dolezalova, Bas Vastert, Nicola Ruperto, Nico Wulffraat

**Affiliations:** 1 Paediatric Rheumatology Unit, 1st Faculty of Medicine, Charles University in Prague and General University Hospital in Prague, Prague, Czech Republic; 2 Department of Pediatric Rheumatology, University Medical Center Utrecht, Utrecht, Netherlands; 3 Pediatria II, Reumatologia, PRINTO, Istituto G. Gaslini, Genova, Italy

## Introduction

A need for evidence-based guidelines for the management of paediatric rheumatic diseases (PRD) has been recognised. In 2012 the European Agency for Health and Consumers signed the contract for a new European initiative SHARE: Single Hub and Access point for paediatric Rheumatology in Europe (project number 2011 1202).

## Objectives

Workpackage 4 (WP4) aimed to identify current access to paediatric rheumatology services and practice in European countries in their complexity. Non-European countries from the PRINTO network were included in order to allow comparisons.

## Methods

The survey questionnaires were developed through consensus meetings of the SHARE consortium partners and consultations with experts from across PReS Working Groups. The Surveys have been stratified into 4 levels: Country-wide, Centre-specific, Disease-specific and Personal. Final survey texts were transformed into electronic web-based system hosted by PRINTO and presented to paediatric rheumatology community via PRINTO mailing list. Results of the country survey are presented here.

## Results

The country survey was completed by representatives of 22 European countries (14 Western and 8 Eastern/Central) and 11 other countries. Results were grouped into 3 specific topics: 1.The healthcare system (providers), organisation of paediatric rheumatology (PR) care, access to care. 2.Evidence based and qualified care. 3.Education and employment issues. In majority of European countries health professionals in PR care form an official working group or national society. Proportion of PR patients treated by general paediatricians or adult rheumatologists is low across Europe (mostly ˂25%). Proportion of patients treated in tertiary centres of university hospitals is higher in Eastern European countries. In Western Europe the similar proportion of patients are treated in paediatric departments of other than tertiary hospitals. National guidelines/recommendations are more frequently available in Western than Eastern European countries (64 vs 25%). While licenced biologics are available in all Western countries, this is not the case for Eastern Europe. Moreover, treatment limitations are more prominent in Eastern countries. Official recognition of paediatric rheumatology as a subspecialty varies. It is generally lower in Europe in comparison to non-European countries and reaches 37 (Eastern Europe) to 57% (Western Europe). Defined training scheme is available in over 60% of Western countries but only in 37% of Eastern ones. Availability of educational resources has been good for over 70% of all European countries. The similar responses between Eastern and Western Europe were also related to the availability of PR pre-graduate teaching. Only 35% of Western European countries employ a system of post-graduate/continuing education while such a system works in nearly 90% of Eastern European countries. Over 70% of all respondents have confirmed the need for more paediatric rheumatologists in their countries within the next 5 years.

## Conclusion

Through the WP4 of the SHARE project important information has been collected forming a thorough inventory of paediatric rheumatology practice in individual European countries. This will serve as a baseline for the formulation of internationally achievable best practices in PRD management. These practices will be presented to stakeholders such as individual paediatric rheumatology units, health authorities, health insurance companies and patient/parent organisations.

## Disclosure of interest

None declared

